# Air-to-Air Heat and Moisture Recovery in a Plate-Frame Exchanger Using Composite and Asymmetric Membranes

**DOI:** 10.3390/membranes12050484

**Published:** 2022-04-29

**Authors:** Amir Jahed Mogharrab, Seyedmehdi Sharifian, Neda Asasian-Kolur, Ali Ghadimi, Bahram Haddadi, Michael Harasek

**Affiliations:** 1 Fouman Faculty of Engineering, College of Engineering, University of Tehran, Fouman 43516-66456, Iran; amir.jahed.m@ut.ac.ir (A.J.M.); s.m.sharifian@gmail.com (S.S.); 2 Institute of Chemical, Environmental and Bioscience Engineering, Technische Universität Wien, Getreidemarkt 9/166, A-1060 Vienna, Austria; bahram.haddadi.sisakht@tuwien.ac.at (B.H.); michael.harasek@tuwien.ac.at (M.H.); 3 Faculty of Petrochemicals, Iran Polymer and Petrochemical Institute, Tehran 14965-115, Iran; a.ghadimi@ippi.ac.ir

**Keywords:** enthalpy exchanger, asymmetric porous membrane, thin film composite membrane, moisture and heat transfer, effectiveness

## Abstract

The present work studied an air-to-air exchanger comprising a flat plate module with a diagonal channel and a counterflow configuration for the air streams. The objective of this study was to remove moisture and sensible heat from an exhaust air stream by indirect contact with another air stream. The temperature and flow rate of the exhaust air was in the range of 40–80 °C and 1–5 L·min^−1^, respectively, and the fresh ambient air to exhaust air flow ratio was 1–5. An asymmetric porous membrane (P-MEM), a thin film composite membrane (C-MEM), and a kraft paper were used as the core for the heat exchange module. The most influential parameter was the humid air temperature, with a direct positive effect (50–60%) due to the increase in the kinetic energy of the water molecules. The other effective parameter was the flow rate of the humid gas with a reverse effect on the enthalpy exchanger performance (25–37%). The ratio of “fresh” air to “exhaust” air had the lowest positive effect (8–10%) on the total effectiveness. The sensible effectiveness of different membranes under the studied conditions was relatively the same, showing their similar heat conductivity. However, the kraft paper showed the best performance compared to the synthetic membranes due to having a porous/hydrophile texture. P-MEM with an asymmetric porous texture showed the closest performance to kraft paper. Furthermore, it was found that under limited conditions, such as higher temperatures (70 and 80 °C) and flow rates (5 L·min^−1^) for the humid air, the performance of P-MEM was a little better than the kraft paper. However, C-MEM with the lowest total effectiveness and overall heat transfer coefficient (150–210 W·m^−2^·K^−1^) showed that the hydrophile PEBAX layer could not contribute to moisture recovery due to its high thickness.

## 1. Introduction

Considering the adverse effects of a humid environment on human health for people who spend a lot of time indoors, reducing humidity and ventilation are constant issues in households [1]. Humidity at high temperatures plays a vital role in problems ranging from mild skin discomfort to more severe issues, such as creating a suitable environment for microbes and viruses to grow and shortening equipment lifetime. In addition, HVAC systems operating in humid weather consume more energy than in dry weather to provide the same cooling capacity and dissipate the latent energy of water vapor molecules [2].

Conventional vapor compression refrigeration systems used in heating, ventilation, and air conditioning (HVAC) applications suffer from high energy demand, high operating cost, excessive cooling of dry air, and providing uncomfortable living conditions, especially at highly humid air streams [3]. Air-to-air energy recovery systems were developed to recover both sensible and latent heat from moist air and use energy from room exhaust air to preheat or precool fresh air before entering the air conditioning system. All these reduce HVAC system operating costs [4]. Among the various energy recovery systems, membrane-based plate and frame enthalpy exchangers have advantages such as a compact structure, easy installation and maintenance, no moving parts, and no crossovers. In addition, membrane-based enthalpy exchangers provide conditions to continuously achieve the desired humidity and sensible heat recovery without regeneration time [5].

Plate and frame membrane heat exchangers consist of parallel, stationary plates that separate different gas flow channels by thin plates. The main difference between membrane enthalpy exchangers and sensible heat exchangers is the material of the separating plates; in these systems, thin semipermeable membrane layers are used instead of metal plates. Semipermeable membranes provide a contact surface for mass (moisture) and heat transfer between two streams of exhaust air from buildings (humid) and fresh ambient air (dry) through the membrane [6]. The flow configurations in plate membrane heat exchangers are co-current, counter-current, cross-current, and mixed flow. The performance of membrane-based enthalpy exchangers depends on heat transfer due to the temperature difference between the gas streams on both sides of the membrane and moisture transfer due to the vapor partial pressure difference between the humid air and the dry air.

One of the most influential parameters for the dehumidification properties of membrane-based heat exchangers is the membrane material, its permeability to water vapor molecules, and its selectivity to prevent other molecules from passing through the membrane. Various types of membranes with different materials and textures have been used as the core for enthalpy exchangers. An earlier study by the authors contains a comprehensive examination of the various types of membranes for this purpose [7]. Kraft papers are traditional membrane cores for dehumidifying air with relatively high efficiency. However, they have weaknesses, such as short durability and the ability to grow bacteria [8]. Nasif et al. evaluated the efficiency of 60 and 70 g m^−2^ kraft paper as the core for the membrane-based heat exchanger using a quasi-counter current and pointed out the significant impact of membrane mass transfer resistance on system performance [9,10].

The best known porous hydrophilic polymer membranes proposed to be used for air dehumidification are ethyl cellulose (EC), polyvinyl alcohol (PVA), cellulose acetate (CA), polyimide (PI), Polyether block amide (PEBAX), and sulphonated poly (ether ether ketone) (SPEEK) [11,12,13,14]. Zhang et al. compared the performance of three different membrane materials, i.e., kraft paper, cellulose acetate (CA), and the modified CA, as the core for the membrane heat exchanger at a steady state. The experiment showed that different membrane materials, thickness, and operating conditions affect latent efficiency while sensible efficiency does not experience any significant change. The modified CA showed the highest latent efficiency among these three membranes [15].

Most unmodified single polymer membranes are mechanically unstable despite acceptable and, in some cases, high permeability. On the other hand, some with sufficiently high mechanical stability do not have sufficient water permeability [14]. Therefore, many authors propose using asymmetric composite membranes containing a porous support layer and a hydrophilic active layer with a smaller pore size. Zhang et al. synthesized a novel vapor-permeable PVA/LiCl membrane for air dehumidification. The membrane consisted of a porous polyethersulfone (PES) support layer and a dense polyvinyl alcohol (PVA) active layer. The PVA solution was modified with LiCl as an additive to facilitate moisture permeation. The addition of LiCl increased the hydrophilicity of the membrane and decreased its crystallinity, making it more flexible and mechanically robust [16]. Hydrophilization of a porous polypropylene membrane with poly(acrylamide-co-acrylic acid) (PAMAC) was performed by Roy et al. High sorption of water vapor of nearly one gram per gram of membrane was achieved by H-bonding with functional groups of PAMAC [17]. Continuous dip coating of porous PVDF membranes with thin PVA layers was carried out by Jesswein et al. to produce membranes for humidification for use in polymer electrolyte fuel cells. Thicker coatings were found to have higher water vapor permeability, which could be due to a lower degree of crosslinking [18]. A composite of PEI and PDMS was also prepared by Kneifel et al. In this case, an adverse effect of the coating on permeability was found, which was minimized by reducing the coating thickness [19]. 

Zhang et al. proposed a one-step preparation of asymmetric cellulose acetate membrane for air-to-air energy recovery. With an environmentally friendly and simultaneous procedure using the wet-phase inversion method, they fabricated membranes with high moisture permeability and exclusion of CO_2_. Increasing the additive content in the casting solution changed the porous texture of the membrane from symmetric to asymmetric [20]. Al-Waked and Nasif conducted CFD modeling and experimental studies to determine the performance of different membranes such as kraft paper (45 and 60 g m^−2^), modified cellulose acetate membrane, and PVA/LiCl mixed membrane. It was found that the heat exchanger with the modified cellulose acetate membrane had the highest energy recovery. The main influencing factor on the performance was the variation of ambient relative humidity [21]. In addition to integral asymmetric membranes with a dense skin layer, thin-film composite membranes are highly-specific membranes that have been proposed for water treatment, pervaporation, and gas/vapor separation [22,23]. However, their performance in air-to-air energy recovery has been studied very limitedly [24]. 

In addition to the membrane type, the flow configuration within the module is another critical parameter. Various flow configurations have been studied, including co-current, cross-current, counter-current, and mixed flow, and it has been shown that the highest performance is obtained with the counter-current configuration. However, some advantages, such as ease of isolation and handling, are reported for the cross-flow and mixed-flow configurations [15]. Other factors, such as changing the channel shapes by using corrugated membranes in each channel and using baffles, have been investigated in various studies to increase the turbulence of the flow and improve heat and moisture transfer [25]. 

Other aspects of the operation of membrane-based air-to-air energy exchangers have also been studied, such as the effect of fouling of particles, which was investigated by Engarnevis et al. for fine and coarse particles as well as ultrafine aerosols. The membrane core was commercial, a dense hydrophilic copolymer film deposited on a polyethylene-based microporous substrate. They found that the coarse dust loading that can occur when the membrane is exposed to a heavily polluted environment for several years has minimal effect on performance. Furthermore, the deposition of particles in dry air only matters if the fouling is severe enough to form a cake layer on the membrane surface comparable to the thickness of the membrane [26].

In the present work, two types of asymmetric composite membranes were fabricated and used in a laboratory-scale enthalpy exchanger to investigate their performance and compare them with a kraft paper. Similar membranes have not yet been studied for this purpose. The heat exchanger consisted of a flat plate module containing a diagonal channel with a counterflow configuration. In the present work, the counterflow configuration was chosen due to its higher efficiency compared to other flow configurations. This comparison was made under different operating parameters such as temperature gradient, flow rate, and flow ratio to determine the energy recovery efficiency of the heat exchanger.

## 2. Experimental

### 2.1. Exchanger Setup

**Figure 1:**
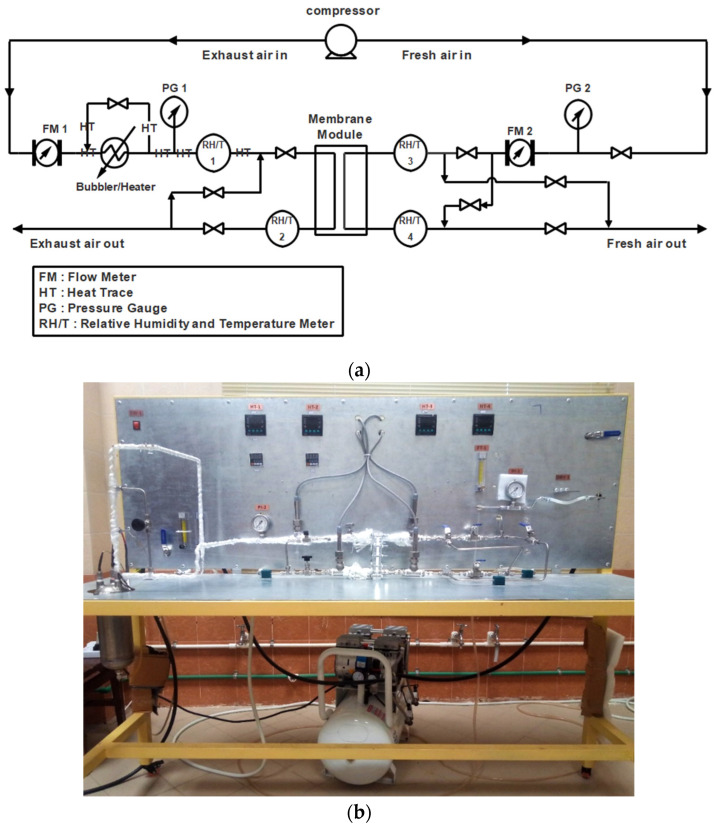
(**a**) Schematic flow diagram and (**b**) the experimental setup of the membrane-based heat exchanger for air dehumidification.

Figure 1a shows a schematic diagram of the membrane-based heat exchanger used in this study. As shown, both the “exhaust” and “fresh” air streams are supplied by a compressor. An inline heater preheats the “exhaust” air stream. After its flow is adjusted with a flow meter, it enters a water-containing bubbler equipped with a thermocouple that allows the operator to adjust the temperature and relative humidity of the “exhaust” air. Behind the bubbler is a bypass to control the relative humidity of the exhaust air as needed. A heat-tracing system further heats the humid air leaving the bubbler to maintain its temperature and prevent vapor condensation. The air streams’ pressure, humidity, and temperature are measured before they enter the membrane module. A counter-current arrangement for the gas streams was considered in the membrane module. After transferring heat and humidity from the “exhaust” to the “fresh” air, the outlet streams were subjected to temperature, humidity, and pressure gauges. The inlet and outlet paths are isolated to obtain more accurate measurements.

Figure 1b shows the experimental laboratory setup used for this study. In this setup, the air flow rates can be varied in the range of 0.5 to 5 L·min^−1^, which allows different flow conditions. As mentioned earlier, the temperature of the incoming moist air (“exhaust”) from the bubbler can be adjusted before it enters the module. Table 1 shows the specifications of the various devices installed.

**Figure 2:**
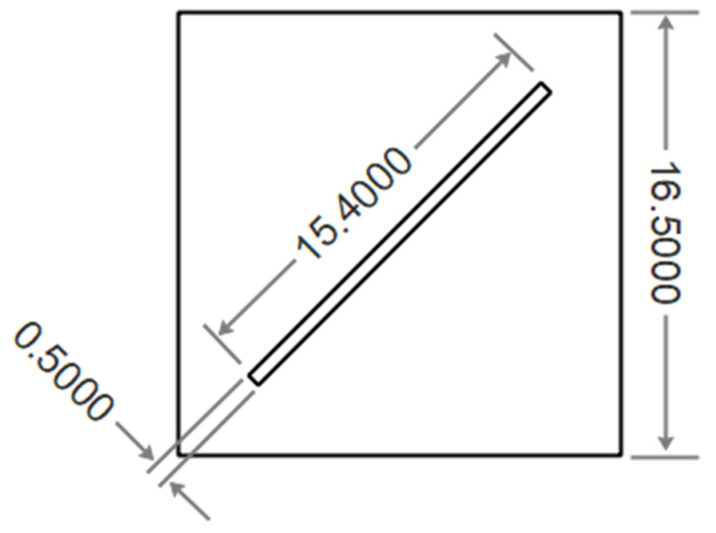
Schematic diagram of the flat membrane module with a diagonal channel for the air flow (dimensions in cm).

Uncertainties in the measurement of data from various instruments used in our setup are as follows: temperature and humidity sensor, ±0.5%; pressure gauge, ±1.0%; and airflow, ±2.5%, giving a total measurement uncertainty of ±4%. As shown in Figure 2, the membrane module designed in this study is a flat plate with a single diagonal channel for gas flow (depth, 0.6 cm; width, 0.5 cm; and length, 15.4 cm). The container is made of Plexiglas^®^ (polymethyl methacrylate) with a Length of 16.5 cm. Plexiglas^®^ is an excellent choice to prevent water absorption and heat transfer through the body due to its hydrophobicity and low thermal conductivity.

The procedure of experiments is as follows: in order to obtain the inlet “exhaust” air, the air flow supplied by the compressor is directed into the bubbler. The inlet “exhaust” air into the membrane module will have a predetermined humidity depending on the water and air temperature. The relative humidity of the inlet and outlet air streams (fresh air and exhaust air) was measured using relative humidity and temperature sensor/indicator (ENDA EHTC7425A) (Table 1). The conversion of relative humidity to absolute humidity was also performed at a known temperature. The humidity/temperature sensors were installed as close as possible to the membrane module to minimize errors due to possible moisture condensation. In addition, the pipes were perfectly insulated in this way to avoid heat loss and temperature differences. The “fresh” air, which should be in contact with the “exhaust” air, is also supplied by the compressor, but has ambient temperature and humidity. During each test, these two streams are fed into the module and come into contact with each other in a counter-current process. The temperature and relative humidity of the “exhaust” and “fresh” air streams are measured and noted until a steady-state is reached. The relative humidity and temperature of the streams in the steady-state are used to determine different types of effectiveness. In addition to temperature and humidity, the air streams’ volumetric flow rate and pressure were measured and recorded before entering the bubbler (on the hot side) and before entering on the cold side of the membrane.

Three series of experiments were conducted with the operating parameters listed below:

(1) Gas stream flowrate: it was varied in 1–5 L·min^−1^. In this series of experiments, the ratio of “fresh” air to “exhaust” air was equal to 1, and the humid supply air flow temperature was 50 °C;
(2) The ratio of fresh air to exhaust air flow rate: it was studied in the range of 1 to 5, while the temperature and flow rate of the incoming “exhaust” air was kept constant at 50 °C and 1 L·min^−1^, respectively;
(3) Humid air temperature: its effect was determined by varying between 40 and 80 °C. In contrast, the ratio of fresh air to exhaust air was equal to 1, and the “exhaust” air flow rate of 2 L·min^−1^ was considered.

In all tests, the incoming “fresh” air’s temperature and relative humidity were adjusted to the ambient conditions. The tests were performed with three types of membranes, which are explained in the Section 2.2 (kraft paper, P-MEM, and C-MEM).

### 2.2. Membrane Cores Preparation and Characterization

In the present study, the performance of three types of membrane cores was compared in both sensible and latent heat exchange. The first membrane type was a 70 g m^−2^ kraft paper from a local company (Pars Paper, Iran). It was applied without any modification as a reference membrane to compare the performance of two synthetic membranes. As mentioned earlier, paper membranes are the classic/traditional membranes used in air-to-air energy recovery systems due to their low cost, availability, and ease of use [7,8].

According to the following procedure, two other synthetic membranes (P-MEM and C-MEM) were prepared using the materials listed in Table 2. The synthetic membranes include an asymmetric porous membrane prepared from polyethersulfone (PES) and a thin film composite membrane prepared by coating PES porous support with PEBAX-1657. The porous supports (P-MEM) were prepared on the nonwoven web using the solution casting process through the non-solvent induced phase separation (NIPS) method. The casting solutions were prepared with N, N-dimethylformamide (DMF) as the solvent and PES concentration of 23%. Some of the main specifications of the PES supports are listed below: average pore radius, 59 nm; effective surface porosity (*ε*/q^2^), 0.00942%; skin thickness, 0.886 µm; and membrane surface porosity (*ε*), 0.4. As mentioned above, the composite membranes (C-MEM) were prepared by the dip-coating method using EtOH/water mixture (70:30 *w/w*) as PEBAX solvent. The coating solution was prepared with a polymer concentration of 3 wt%. More detailed information about the membrane preparation procedure can be found in our previous work [24,27].

**Figure 3:**
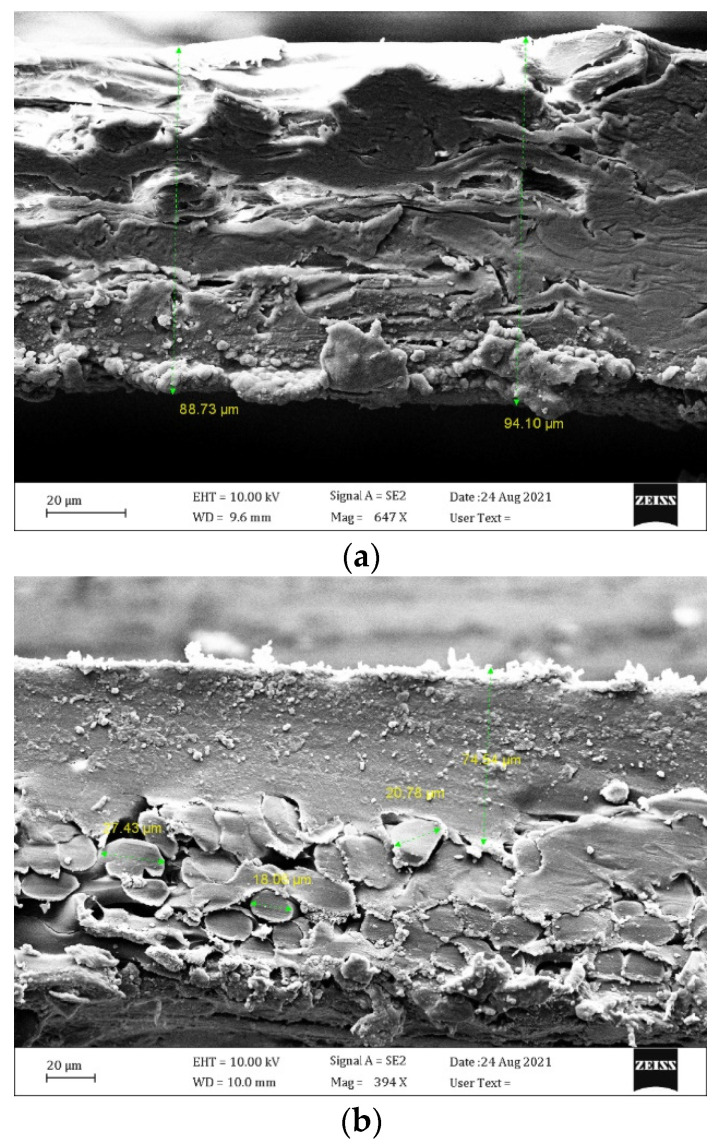

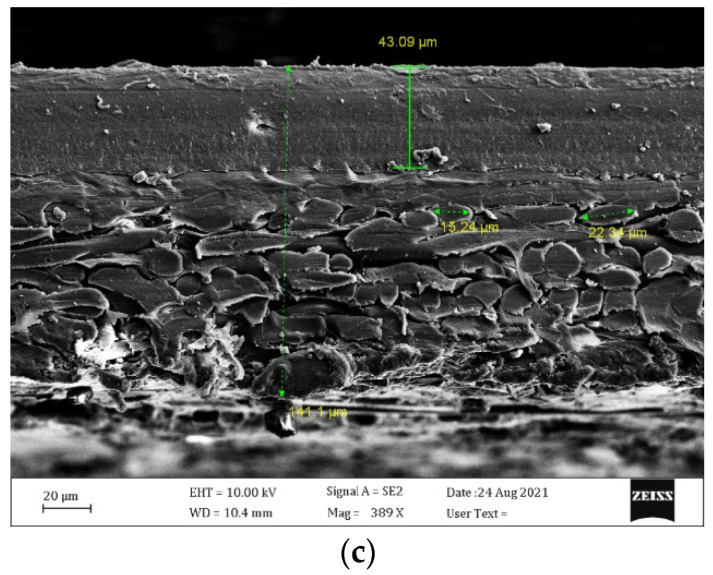
FESEM micrographs of the cross-section of (**a**) kraft paper, (**b**) P-MEM, and (**c**) C-MEM.

A Sigma VP FESEM microscope (Zeiss, Munich, Germany) was used to acquire FESEM images of the cross-section of these membranes. The microscopic diagrams in Figure 3 show the asymmetric texture of P-MEM and C-MEM, both with porous mechanical support on the underside. C-MEM has a fragile smooth surface of PEBAX polymer on the top cover. PEBAX is applied as a non-porous, hydrophilic, dense layer on top of the base layers. P-MEM also has a PES coating layer on the base. In contrast, kraft paper has a symmetrical porous texture.

### 2.3. Performance Evaluation 

The amounts of heat and moisture transfer through the membrane at different operating parameters were evaluated using various ratios, including sensible, latent, and total efficiencies. The temperature, pressure, and relative humidity of the incoming and outgoing air streams were measured after the system reached a steady state for these calculations. 

The sensible efficiency (*ε*(*s*)) is the ratio of the sensible heat transfer rate to the maximum possible heat transfer rate due to the temperature difference between the incoming “exhaust” air and the “fresh” air stream; the simplified relationship can be shown as follows (Equation (1)) [28]:

(1) $$\varepsilon \left(s\right)=\frac{\left|T_{E,in}-T_{E,out}\right|}{\left|T_{E,in}-T_{F,in}\right|},$$

where *T* is the absolute temperature (K), and the subscripts *E*, *F*, *in*, and *out* represent the “exhaust” air flow, the “fresh” air flow, the inlet flow, and the outlet flow, respectively.

The latent efficiency (*ε*(*l*)) depends on the amount of mass (humidity) transferred and gives the ratio of latent heat transferred to the maximum possible heat transfer rate between the “exhaust” and the “fresh” air at the inlet due to the humidity difference. The simplified correlation is shown in Equation (2) [28]:

(2) $$\varepsilon \left(l\right)=\frac{\left|{𝓌}_{E,in}-{𝓌}_{E,out}\right|}{\left|{𝓌}_{E,in}-{𝓌}_{F,in}\right|},$$

where 𝓌 is the humidity ratio (kg _H2O_/kg _dry air_).

The total effectiveness (*ε*(*t*)) is essentially the enthalpy difference between the “exhaust” air at the inlet and the outlet to the enthalpy difference between the “exhaust” air and the “fresh” air at the inlet. This relationship can be represented by Equation 3 [28]:

(3) $$\varepsilon \left(t\right)=\frac{\left|{𝓀}_{E,in}-{𝓀}_{E,out}\right|}{\left|{𝓀}_{E,in}-{𝓀}_{F,in}\right|},$$

where 𝓀 (kJ/kg _dry air_) is the humid enthalpy.

Equations (4)–(6) show the relationships required to calculate the overall heat transfer coefficient (U: W·m^−2^·K^−1^) based on the LMTD method.

(4) $$U=\frac{Q_{t}}{\Delta T_{LMTD}\cdot A}$$

(5) $$Q_{t}={\dot{m}}_{E,in}\left|{𝓀}_{E,in}-{𝓀}_{E,out}\right|$$

(6) $$\Delta T_{LMTD}=\frac{\left(T_{E,in}-T_{F,out}\right)-\left(T_{E,out}-T_{F,in}\right)}{ln\left(\frac{T_{E,in}-T_{F,out}}{T_{E,out}-T_{F,in}}\right)}$$

where $Q_{t}$ (kJ·s^−1^) and $\Delta T_{LMTD}\;$(K) are the total heat transfer rate and the logarithmic mean temperature difference, respectively. Log mean temperature difference is a parameter used to calculate the driving force of heat transfer in flow systems, especially heat exchangers. It gives the logarithmic average of the temperature difference between the hot and cold flows at each end of the heat exchanger.

## 3. Results and Discussion

### 3.1. Error Calculation

Despite using a low conductivity material (Plexiglas^®^) for the membrane module and the insulation of the inlet and outlet air ducts and the module itself, there is a possibility that a small amount of heat will be released from the system to the room environment. It is due to insufficient insulation, which may mean that not all moisture removed from the humid air is released to the “fresh” air stream. These problems can lead to some calculation errors. Since the moisture transfer would transfer a significant amount of heat from the “exhaust” air to the “fresh” air, the error calculation was based on the moisture difference between the incoming and outgoing air streams. The average error percentages of each series of experiments with different membrane cores, kraft paper, P-MEM, and C-MEM are shown in Table 3. It can be seen that the average error percentage is less than 20% in all series and less than 15% in most cases.

### 3.2. Effect of Flow Rate 

**Figure 4:**
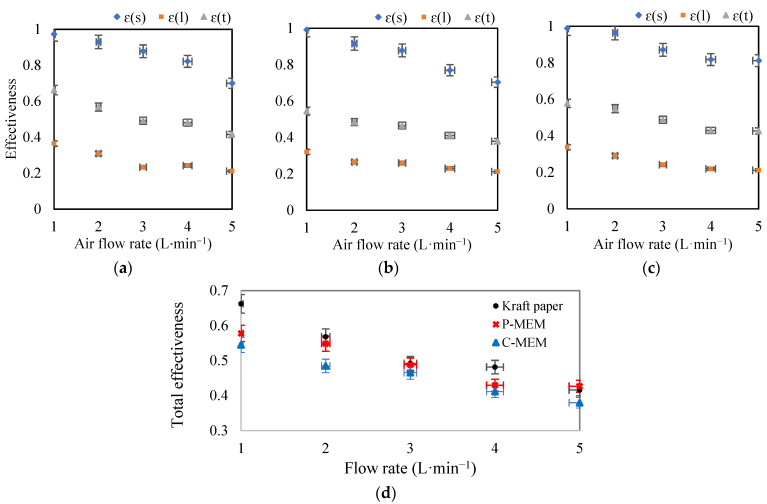
Effect of increasing air flow rates on sensible, latent, total effectiveness for (**a**) kraft paper, (**b**) C-MEM, and (**c**) P-MEM, and (**d**) comparison of the total effectiveness of different membranes at different air flow rates.

The first parameter that affects the performance of a membrane-based heat exchanger is the flow rate of the air streams on both sides of the membrane. This parameter was studied by varying the air flow rate on both sides between 1 and 5 L·min^−1^ when the flow ratio of “exhaust”/”fresh” air was equal to one. This flow rate resulted in laminar flow in the module channel with Reynolds number in the range of 100–500. Figure 4 shows the sensible, latent, and total efficiency of the membrane-based heat exchanger used in this work for three different membrane cores. Under all conditions, the value of the sensible effectiveness was larger than the latent effectiveness, and the total efficiency was between these two values. The lower values of latent efficiency can be attributed to the high resistance of the membranes during mass transfer.

Increasing the volumetric flow rate of both input streams (“exhaust” and “fresh” air) in the range of 1 to 5 L·min^−1^ resulted in a shorter contact time of the air streams with the membrane surface, reducing moisture and heat transfer. This effect decreased the sensible, latent, and total efficiencies, as shown in Figure 4. As can be seen, increasing the flow rate from 1 to 5 L·min^−1^ changed the sensible thermal efficiency in the range of 0.97 to 0.70, 0.99 to 0.70, and 0.99 to 0.81 for the kraft paper membrane, C-MEM, and P-MEM, respectively. The data show that the flow rate significantly affects sensible heat efficiency for all three membranes.

The latent heat recovery by the three membrane cores was relatively the same; *ε*(*l*) for kraft paper, C-MEM, and P-MEM ranged from 0.36–0.21, 0.32–0.21, and 0.34–0.21, respectively. As can be seen, kraft paper showed better moisture transfer at low flow rates, but at higher flow rates, the performance of P-MEM was comparable to that of kraft paper.

Figure 4d compares all three membranes’ overall enthalpy recovery performance at different flow rates. C-MEM showed the weakest performance in latent heat recovery due to its top PEBAX layer. P-MEM as an asymmetric membrane with porous support and a hydrophilic PES texture showed relatively reasonable latent recovery. However, both performances were lower than that of kraft paper. The close values of the total effectiveness of the kraft membrane and P-MEM at high flow rates can be seen in this curve. The authors believe this behavior can be related to moisture transfer mechanisms through the kraft paper and the synthesized P-MEM. The dehumidification ability of kraft paper is mainly due to its water adsorption ability, which is more pronounced at lower flow rates. On the other hand, at higher flow rates, where the porous structure of kraft paper is saturated with water, its water transfer efficiency would decrease.

### 3.3. Effect of Flow Rates Ratio

**Figure 5:**
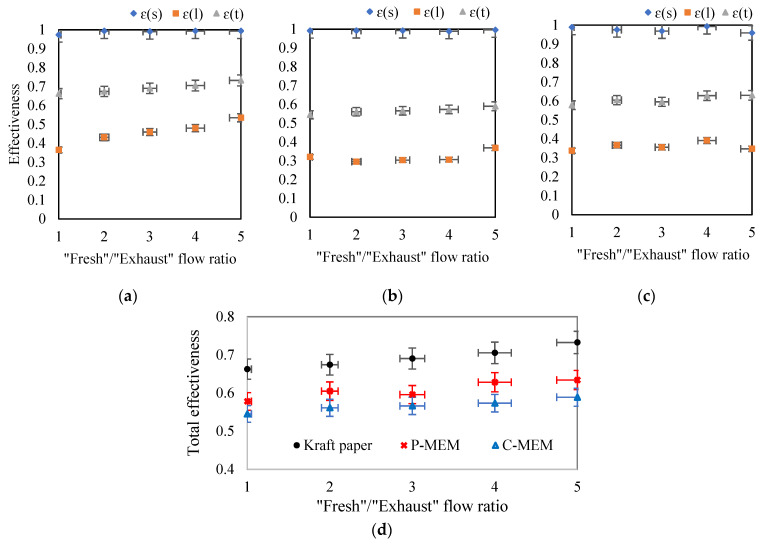
Effect of increasing the flow rate ratio of “fresh” to “exhaust” on sensible, latent, total effectiveness for (**a**) kraft paper, (**b**) C-MEM, and (**c**) P-MEM, (**d**) comparison of the total effectiveness of different membranes at different fresh/exhaust air flow ratios.

The “fresh” air provides the driving force for removing mass and heat from the moist and hot “exhaust” air. Therefore, the flow ratio of these two air streams can be considered a factor affecting the performance of the membrane heat exchanger. Figure 5 shows the effect of increasing the proportion of “fresh” to “exhaust” air from 1 to 5 on the performance of the enthalpy exchanger when the flow rate of the incoming moist “exhaust” air was constant at 1 L·min^−1^. Figure 5 shows that the ratio of the flows has no significant effect on the driving force for sensible heat recovery. This insignificant influence may be related to the relatively low moisture supply by the “exhaust” air, which can be removed efficiently by the “fresh” air with the lowest flow rate. Furthermore, when comparing the three types of membranes (kraft paper, C-MEM, and P-MEM), no significant difference was found in their sensible heat recovery performances. This can be attributed to the similar and negligible heat transfer resistance of membrane cores.

The flow rate ratio is expected to have a more significant effect on latent heat recovery than sensible heat recovery because the higher the flow rate of fresh air, the more water can be swept through the membrane. Kraft paper exhibited the highest *ε*(*l*) under these operating conditions, and P-MEM was intermediate between kraft paper and C-MEM in latent heat effectiveness.

Figure 5d also shows the overall effectiveness of the three different membranes in comparison. There is a relatively small increase in total effectiveness over the entire range of flow ratios (1–5). From the comparison of Figure 4d and Figure 5d, it can be seen that the ratio of “fresh”/”exhaust” air (in the range studied) was not as important as the air flow rate.

### 3.4. Effect of the Humid Air Temperature

**Figure 6:**
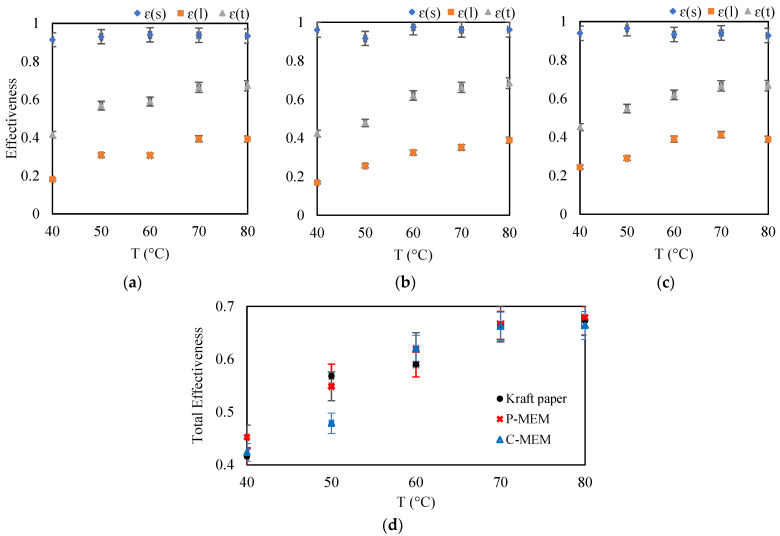
Effect of increasing the incoming humid air temperature on sensible, latent, total effectiveness for (**a**) kraft paper, (**b**) C-MEM, and (**c**) P-MEM, and (**d**) comparison of the total effectiveness of different membranes at different incoming humid air temperatures.

The effect of the temperature of the incoming humid air was studied by varying this parameter between 40 and 80 °C, at a gas flow rate of 2 L·min^−1^ and a ratio of “fresh” to “exhaust” air flow of 1. At higher temperatures, a positive effect on the performance of the membrane-based heat exchangers is expected due to the more significant driving force between two air streams on either side of the membrane. Figure 6a–c shows that the range of change in sensible efficiency for all membranes is between 0.9 and 1. However, there is no significant change in the curves of the sensible efficiencies for the different membranes, which could be evidence of the membranes’ relatively similar and negligible thermal conductivity. 

The latent thermal efficiency increased for all membranes when humid air entered at a higher temperature at constant absolute humidity. A higher temperature increases the molecular energy of the water molecules (vapor) and the diffusion coefficient, which increases the latent effectiveness. The overall efficiency or enthalpy recovery rate also shows the same trend with increasing inlet temperature (Figure 6d). In this series of experiments with a relatively high flow rate of 2 L·min^−1^ and high temperature, the performance of the kraft paper deteriorated a little compared to the synthesized P-MEM. It means that P-MEM performs better than the kraft paper under more severe conditions when higher moisture needs to be removed from the “exhaust”.

### 3.5. Overall Heat Transfer Coefficient 

The overall heat transfer coefficients were estimated for the membrane modules based on three different cores, and the average results for each series of tests are shown in Table 4. As can be seen, the magnitude of the overall heat transfer coefficient for the membrane-based enthalpy exchanger used in the present work to recover moisture and heat from the air stream was in the range of 150–250 W·m^−2^·K^−1^ under the operating conditions studied. This overall heat transfer coefficient was more significant for the kraft paper membrane than for the P-MEM and larger than for the C-MEM. It can be attributed to the greater thickness of the synthetic membranes compared to the kraft paper. A direct relationship between the overall heat transfer coefficient (U) and the air flow rate was found among the operating parameters studied for all three membrane cores. As expected, an increase in “exhaust” air flow rate directly affects the overall heat transfer coefficient, which can be attributed to the increase in turbulence and higher mass and heat transfer rates.

## 4. Conclusions

The study of the factors affecting the efficiency of air-to-air enthalpy exchangers is the main objective of the present work. Three different types of membranes, including a 70 g·m^−2^ kraft paper, an asymmetric porous membrane (P-MEM), and a thin-film composite membrane (C-MEM), were used in a plate-and-frame air-to-air enthalpy exchanger. Energy recovery experiments were conducted to investigate the effects of humid air flow rate (1–5 L·min^−1^), fresh air to exhaust airflow ratio (1 to 5), and humid air inlet temperature (40 to 80 °C). The main results of the present work are as follows: 

The total effectiveness of the membrane heat exchanger with these three types of membranes ranged from 0.38–0.74.

The best performance, especially at low humid gas flow rates and low moisture loading, was obtained with the kraft paper, which is related to its higher water adsorption capacity. 

At higher flow rates and higher moisture content of the humid gas, P-MEM showed better performance. 

The PEBAX layer on the surface of C-MEM proved to be an obstacle to moisture transport under all conditions. 

Despite the positive effect, the ratio of dry to moist air flow rate proved to be the least effective parameter for effectiveness under the conditions used. 

Increasing the wet gas flow rate decreased the residence time and thus the effectiveness. 

Increasing the temperature of the inlet humid gas at constant humidity increased the kinetic energy of the molecules and resulted in faster transport. 

The average overall heat transfer coefficient of the system was calculated to be 150 to 250 W·m^−2^·K^−1^, with the highest value for kraft paper and then for P-MEM.

Based on these findings, C-MEM (thin-film composite membrane) is not suitable for water vapor transfer and energy recovery in air-to-air enthalpy exchangers. Considering the hydrophilic structure of PEBAX, the hindering factor is the relatively large thickness of this layer, which cannot ensure fast water transfer between two hot and cold sides. Therefore, in addition to the high water permeability, the transfer rate should also be considered in further studies. The most efficient was the kraft paper with a porous structure, which ensures a high adsorption rate and high affinity to H_2_O due to its hydrophilic texture. P-MEM as an asymmetric porous membrane showed relatively good performance, whose structure can be optimized to obtain better results.

## Figures and Tables

**Table 1:** The main components of membrane-based heat exchanger setup with detailed specification.

Main Segments	Equipment	Specification
Air supply	Air compressor	Active AC1350S, 50 L
Humidifying column	Cylinder material, dimensions, Gas nozzle, position Water level	SS 304, thickness: 4 mm, volume: 2 L Swagelok^®^ SC-11 gas filter, 3 cm above the bottom of column 1 L
Membrane module	Channel width × height × length Material Total/Effective surface area	5 mm × 6 mm × 15.4 cm Plexiglas^®^ 7.7 cm^2^
Instruments	Air flow meter Heat trace device Needle valve Ball valve Pressure gauge Relative humidity and temperature sensor/indicator Pipeline	LZB-DK, 0.5–5 L·min^−1^ Silicon rubber heat generation,100 W Parker Hannifin, DE-LOK, SS 316 Parker, SS 316, ¼; Nippon, SS, ¼ Wika, EN 837-1, 0–4 bar ENDA EHTC7425A Stainless steel, ¼

**Table 2:** Materials required for the synthesis of membranes and their properties.

Material	Company
PES Ultrason^®^ E 6020P	BASF (Ludwigshafen, Germany)
PEBA polymer (trade name PEBAX-1657)	Arkema (Colombes, France)
Absolute ethanol (EtOH)	Merck (Darmstadt, Germany)
N,N-dimethylformamide (DMF) [(CH_3_)_2_NC(O)H]	Merck (Darmstadt, Germany)

**Table 3:** The average percentage of error for each set of experiments under different operational conditions.

Variable	Changing Range	Kraft Paper	P-MEM	C-MEM
Air flow rate	1–5 L·min^−1^	12.8	11.1	13.8
“Fresh” to “Exhaust” air flow rate ratio	1–5	19.0	11.7	12.4
Temperature of the inlet “exhaust” air	40–80 °C	13.7	15.3	12.6

**Table 4:** The overall heat transfer coefficient (U) of the membrane-based heat exchanger for three different membrane cores.

Test Series	Operating Range	Overall Heat Transfer Coefficient (W·m^−2^·K^−1^)		
		Kraft Paper	C-MEM	P-MEM
Flow rate	1–5 L·min^−1^ (Flow ratio: 1, T = 50 °C)	252	211	238
Flow ratio	1–5 (Flow rate: 1 L·min^−1^; T = 50 °C)	192	153	185
Temperature	40–80 °C (Flow rate: 2 L·min^−1^; Flow ratio: 1)	224	192	219

## Data Availability

The data presented in this study are available on request from the corresponding author.

## Nomenclature

**Abbreviations**	**Full name**
CA	Cellulose acetate
C-MEM	Thin film composite membrane
DMF	Dimethylformamide
EC	Ethyl cellulose
EtOH	Ethyl alcohol (ethanol)
HVAC	Heating Ventilation and Air Conditioning
LiCl	Lithium chloride
NIPS	Non-solvent induced phase separation
PAMAC	Poly(acrylamide-co-acrylic acid)
PDMS	Polydimethylsiloxane
PEBAX	(PEBA) Polyether block amide
PEI	Polyetherimide
PES	Polyethersulfone
PI	polyimide
P-MEM	Asymmetric porous membrane
PVA	Polyvinyl alcohol
PVDF	Polyvinylidene fluoride
SPEEK	Sulphonated poly (ether ether ketone)
**Symbols/units**	**Explanation**
A (m^2^)	Membrane surface
*ε*(*l*)	Latent efficiency
*ε*(*s*)	Sensible efficiency
*ε*(*t*)	total effectiveness
$\Delta T_{LMTD}$ (K)	Logarithmic mean temperature difference
*𝓀* (kJ/kg _dry air_)	Humid enthalpy
${𝓀}_{E,in}$ (kJ/kg _dry air_)	Humid enthalpy of inlet “Exhaust” stream
${𝓀}_{E,out}$ (kJ/kg _dry air_)	Humid enthalpy of outlet “Exhaust” stream
${𝓀}_{F,in}$ (kJ/kg _dry air_)	Humid enthalpy of inlet “Fresh” stream
${\dot{m}}_{E,in}$ (kg _dry air_/s)	Mass flow rate of inlet dry “Exhaust” air stream
*T* (K)	Absolute temperature
$T_{E,in}$ (K)	Temperature of inlet “Exhaust” stream
$T_{E,out}$ (K)	Temperature of outlet “Exhaust” stream
$T_{F,in}$ (K)	Temperature of inlet “Fresh” stream
$T_{F,out}$ (K)	Temperature of outlet “Fresh” stream
$Q_{t}$ (kJ·s^−1^)	Total heat transfer rate
U (W·m^−2^·K^−1^)	Overall heat transfer coefficient
*𝓌* (kg _H2O_/kg _dry air_)	Absolute humidity (Humidity ratio)
${𝓌}_{E,in}$ (kg _H2O_/kg _dry air_)	Absolute humidity of inlet “Exhaust” stream
${𝓌}_{E,out}$ (kg _H2O_/kg _dry air_)	Absolute humidity of outlet “Exhaust” stream
${𝓌}_{F,in}$ (kg _H2O_/kg _dry air_)	Absolute humidity of inlet “Fresh” stream

## References

[1] Liang C., Zhang L., Pei L. (2009). Independent air dehumidification with membrane-based total heat recovery: Modeling and experimental validation. Int. J. Refrig..

[2] Xu Q., Riffat S., Zhang S. (2019). Review of Heat Recovery Technologies for Building Applications. Energies.

[3] Mardiana-Idayu A., Riffat S. (2012). Review on heat recovery technologies for building applications. Renew. Sustain. Energy Rev..

[4] Ong K.S. (2014). Review of heat pipe heat exchangers for enhanced dehumidification and cooling in air conditioning systems. Int. J. Low-Carbon Technol..

[5] Nasif M.S., Morrison G.L., Behnia M. (2017). Membrane Based Enthalpy Heat Exchanger Performance in HVAC System. J. Appl. Membr. Sci. Technol..

[6] Zhang L., Jiang Y. (1999). Heat and mass transfer in a membrane-based energy recovery ventilator. J. Membr. Sci..

[7] Asasian-Kolur N., Sharifian S., Haddadi B., Pourhoseinian M., Shekarbaghani Z.M., Harasek M. (2021). Membrane-based enthalpy exchangers for coincident sensible and latent heat recovery. Energy Convers. Manag..

[8] Yang B., Yuan W., Gao F., Guo B. (2013). A review of membrane-based air dehumidification. Indoor Built Environ..

[9] Nasif M.S., Al-Waked R., Behnia M., Morrison G. (2013). Air to air fixed plate enthalpy heat exchanger, performance variation and energy analysis. J. Mech. Sci. Technol..

[10] Nasif M., Al-Waked R., Morrison G., Behnia M. (2010). Membrane heat exchanger in HVAC energy recovery systems, systems energy analysis. Energy Build..

[11] Hansen C.M. (1980). Diffusion in polymers. Polym. Eng. Sci..

[12] Allen S., Fujii M., Stannett V., Hopfenberg H., Williams J. (1977). The barrier properties of polyacrylonitrile. J. Membr. Sci..

[13] Metz S., Van De Ven W., Potreck J., Mulder M., Wessling M. (2005). Transport of water vapor and inert gas mixtures through highly selective and highly permeable polymer membranes. J. Membr. Sci..

[14] Narducci R., Di Vona M.L., Marrocchi A., Baldinelli G. (2018). Stabilized SPEEK Membranes with a High Degree of Sulfonation for Enthalpy Heat Exchangers. Coatings.

[15] Zhang L., Liang C., Pei L. (2008). Heat and moisture transfer in application scale parallel-plates enthalpy exchangers with novel membrane materials. J. Membr. Sci..

[16] Zhang L.-Z., Wang Y.-Y., Wang C.-L., Xiang H. (2008). Synthesis and characterization of a PVA/LiCl blend membrane for air dehumidification. J. Membr. Sci..

[17] Roy S., Hussain C.M., Mitra S. (2013). Poly(acrylamide-co-acrylic acid) hydrophilization of porous polypropylene membrane for dehumidification. Sep. Purif. Technol..

[18] Jesswein I., Hirth T., Schiestel T. (2017). Continuous dip coating of PVDF hollow fiber membranes with PVA for humidification. J. Membr. Sci..

[19] Kneifel K., Nowak S., Albrecht W., Hilke R., Just R., Peinemann K.-V. (2006). Hollow fiber membrane contactor for air humidity control: Modules and membranes. J. Membr. Sci..

[20] Zhang X.R., Zhang L.Z., Liu H.M., Pei L.X. (2011). One-step fabrication and analysis of an asymmetric cellulose acetate membrane for heat and moisture recovery. J. Membr. Sci..

[21] Al-Waked R.F., Nasif M.S. (2019). Air to Air Energy Recovery from HVAC Systems under Different Membrane Materials. Univers. J. Mech. Eng..

[22] Khulbe K.C., Matsuura T. (2018). Thin film composite and/or thin film nanocomposite hollow fiber membrane for water treatment, pervaporation, and gas/vapor separation. Polymers.

[23] Abetz V., Brinkmann T., Sözbilir M. (2021). Fabrication and function of polymer membranes. Chem. Teach. Int..

[24] Salafi M., Asasian-Kolur N., Sharifian S., Ghadimi A. (2021). A flat-plate spiral-channeled membrane heat exchanger for methane dehumidification: Comparison of kraft paper and thin-film composite membrane. Int. J. Therm. Sci..

[25] Pourhoseinian M., Asasian-Kolur N., Sharifian S. (2021). CFD investigation of heat and moisture recovery from air with membrane heat exchanger. Appl. Therm. Eng..

[26] Engarnevis A., Huizing R., Green S., Rogak S. (2017). Particulate fouling assessment in membrane based air-to-air energy exchangers. Energy Build..

[27] Ghadimi A., Norouzbahari S., Lin H., Rabiee H., Sadatnia B. (2018). Geometric restriction of microporous supports on gas permeance efficiency of thin film composite membranes. J. Membr. Sci..

[28] Deshko V., Karvatskii A., Sukhodub I.O. (2016). Heat and mass transfer in cross-flow air-to-air membrane heat exchanger in heating mode. Appl. Therm. Eng..

